# Use of Chloride of Soda

**Published:** 1853-10

**Authors:** 


### *Use of Chloride of Soda.*

The efficacy of Chloride of Soda in the treatment of Burns, Wounds, Ulcers, &c., has been so decided in our hands, that we feel impelled to say a few words on the subject in consequence of having, not unfrequently, found it to fail in the hands of others; and having always in such cases ascertained that the solution used was much stronger than that *we* are in the habit of prescribing.

The American Chloride of Soda being very seldom good, we never use any other than the French. The directions usually accompanying Labarraque's bottles are, however, apt to mislead those who look to them as a guide in its use. They state, for example, that "a mixture of one part of the chloride with seven or eight of water has been successfully used in chilblains and burns, and also for bad wounds, especially in sores where mortification has commenced." Now, although so strong a solution may be useful when "*mortification has commenced*," it will almost invariably aggravate the condition of burns and recent wounds. Lisfranc, to whom we are indebted for its use in burns, always recommended a solution of half an ounce in a quart of water,—insisting that the application must be weak enough to give *no pain*, but, on the contrary, to *allay* this. The sudden relief of the pain of burns which attends the application of this weak solution by means of cloths dipped into it, and kept wet, is truly astonishing. Whether the cuticle be removed or not, the relief is equally prompt, and the ultimate cure more rapid than by any other medication. One important effect of the remedy, especially in children, is, that it prevents the extensive suppuration which is itself so fatal in such cases.

As an application, or wash, to recent wounds of a lacerated character and to chronic ulcers, especially of the legs, we use a mixture of the same strength; and it is only in cases tending to mortification that we ever apply it sufficiently strong to produce smarting. In short, if its antiphlogistic effect be desired, it must give *no pain* when applied —whereas, if it be used as a styptic or stimulant, it must be made stronger. We know of nothing more irritating, and consequently injurious, to a burn, new wound, or ulcer, than this solution, when made too strong.—*South. Med. and Surg. Jour.*

